# Mechanical Properties of Epoxy Compounds Based on Unmodified Epoxy Resin Modified with Boric Acid as an Antiseptic

**DOI:** 10.3390/ma17010259

**Published:** 2024-01-03

**Authors:** Anna Rudawska

**Affiliations:** Faculty of Mechanical Engineering, Lublin University of Technology, Nadbystrzycka 36, 20-618 Lublin, Poland; a.rudawska@pollub.pl; Tel.: +48-81-53-84-232

**Keywords:** epoxy resin, epoxy compound, curing agent, modification, antiseptic, mechanical properties

## Abstract

The objective of this study was to compare the selected mechanical properties of epoxy compounds based on an unmodified epoxy resin with those containing an antiseptic as a modifying agent. Experiments were carried out on twelve epoxy compounds made of an epoxy resin based on bisphenol A (BPA) with a basic epoxide amount of 0.48–0.51 mol/100 g. Three curing agents were used: one polyamide (a polyaminoamide curing agent) and two amines (one was an adduct of aliphatic amine and aromatic glycidyl ether, and the other was an adduct of cycloaliphatic amine). The epoxy compounds were modified by adding an antiseptic in the form of powdered boric acid (H_3_BO_3_) in three amounts: 0.5 g, 1.0 g, and 1.5 g. The cured modified and unmodified epoxy compounds were subjected to compressive strength testing and microscopic examination. The experimental results showed that the epoxy compounds containing adduct of aliphatic amine (triethylenetetramine) and aromatic glycidyl ether as the amine curing agent, i.e., E5/ET/100:18, had the highest compressive strength out of all the tested epoxy compounds, with the highest value of 119 MPa obtained for the epoxy compound modified by the addition of 1.0 g boric acid. The epoxy compounds modified with boric acid acquired antiseptic properties and, for most cases, exhibited a higher compressive strength than the unmodified epoxy compounds (not lower than that specified by the manufacturer for unmodified epoxy compounds).

## 1. Introduction

Epoxy resins are most widely used in the production of adhesive materials [1,2,3]. Epoxy adhesives based on epoxy resins [4,5] are universal adhesives that are widely available on the market. Epoxy resins are used in electronics as well as electrical, automotive, marine, aviation, and construction industries [6,7]. Apart from their main application for joining various materials, they are used as basic components of composites, nanocomposites and laminates [7,8,9,10,11], coatings [12,13,14,15,16,17], polymer floors, paints, sealants and impregnates, as well as medical materials characterized by, e.g., antibacterial properties [9,16,18,19,20,21].

To obtain the desired properties of an adhesive, coating, or composite, epoxy resins are subjected to curing [22,23,24,25]. The choice of a curing agent type depends on the desired properties of the epoxy materials [5,26,27,28]. Prolongo et al. [24] tested a new recipe for epoxy systems based on the use of polyaminosiloxane as a curing agent, showing that the developed material was characterized by relatively good adhesion, high γ, and low water absorption when compared to other amine/epoxy resins. Ignatenko et al. [29] investigated the properties of epoxy resins cured with aromatic amines, emphasizing that the use of such curing agents resulted in a very good combination of physical and mechanical properties of the fabricated epoxy materials. Kocaman and Ahmetli [26] analyzed a DGEBA-type modified epoxy resin (ER) using acrylic soybean oil (AESO) cured with various types of curing agents, demonstrating, among others, that the coatings obtained from the modified epoxy resins (M-ER) and cured with anhydride curing agents were highly resistant to corrosive acidic media. Grillet et al. [27] studied the influence of the structure of aromatic curing agents (various aromatic diamines) on the curing kinetics of the epoxy networks of an epoxy prepolymer based on diglycidyl ether of bisphenol A (DGEBA). Ferdosian et al. [28] showed that the thermal stability of cured lignin-based epoxy resins also depended on the types of curing agents and lignin used for the synthesis of bio-based epoxy resins.

The properties of epoxy compounds can be efficiently modified not only by using the right type of a curing agent, but also by adding fillers or other polymers [30] to improve the specific properties of these materials [6,11,20,31]. Mohan [25] reviewed the literature related to the modification of epoxy resins and their properties that are applicable to industrial applications. Brantseva et al. [30] tested the ability of epoxy–anhydride systems modified with up to 8 wt% poly(vinyl acetate) (PVAc) and up to 6 wt% poly(vinyl butyral) (PVB) to improve the crack resistance and impact properties of fabricated epoxy resins. For the epoxy–PVAc mixtures, a 2.4-fold increase in the cracking resistance of the epoxy resin and a simultaneous reduction in its impact strength by up to 30% were observed. For the epoxy–PVB mixtures, the observed increase in the cracking resistance of the epoxy resin was much smaller (45%), but its impact strength increased by 50%.

The modifying substances include fillers in various forms and auxiliaries such as stabilizers, plasticizers, diluents, coloring agents, antipyrines, antiseptics, and antibacterial agents [16,19,31,32,33,34,35]. Boric acid is one of the additives that is used to improve both the fire-resistant properties of polymers (as a flame retardant) and their antiseptic properties (as an antiseptic). Boric acid (H_3_BO_3_) is a weak inorganic acid. It is used both in its pure form and in various chemical compounds [36], and its areas of application include:

(i) industrial processing and production, where it used as a substance for modifying various properties of the main polymeric materials [37,38,39,40,41] as well as epoxy resins [17,42,43], including the flame retardant properties of polymers [44,45,46,47],
(ii) in medicine and cosmetics [48,49], where it is used as a substance with antibacterial properties, as a component of antibacterial protective coatings, and in many medical devices [50].

Studies have shown that boric acid is used as an additive that reduces the flammability of polymers [42,45,51,52,53], e.g., polyurethane or polyurethane–polyisocyanurate foams [44], as well as of epoxy composites [42,43]. Paciorek-Sadowska et al. [44] found that the application of borate as a polyol component and, simultaneously, as a flame retardant in the recipe for the production of PUR-PIR foams was very favorable. The resulting foams were characterized by reduced brittleness, higher compressive strength, and closed cell content, as well as considerably lower flammability in comparison with the standard foam. Visakh et al. [42] investigated the effects of adding different percentages of powdered boric acid as flame-retardant fillers on the thermal properties of the obtained epoxy resin composites. It was shown that the effects of boric acid as a filler depended on its content. Nazarenko et al. [43] studied the influence of boric acid on the volatile products of thermo-oxidative degradation of epoxy polymers, finding that the introduction of boric acid into the epoxy matrix increased the thermal stability of epoxy composites and led to a 2–2.7-fold reduction in toxic gas products. Bagci and Imrek [40] found that the composites containing boric acid in the amount of 15% of the resin showed higher wear than pure materials without filler, which showed that the filler increased the erosive wear resistance of the epoxy resin composites.

Shen et al. [34] emphasized that flame-retardant polymeric materials are vital for use in the construction industry as elements of walls, floors, suspended ceiling cables, and other building equipment elements. In addition, some structural elements also have antiseptic properties. This aspect is currently of great interest due to a growing range of various epoxy composite materials that are available on the market in the form of floors and paints with antiseptic properties. This is particularly important in the construction and building industry, as well as in food industry equipment and medical applications where resistance to microorganisms (bacteria, viruses, and fungi) is essential. Such materials can, among others, be used as antiseptic coatings (containing antiseptic additives) for blocking the development of Gram-positive and Gram-negative bacteria, molds, and fungi on the coating surface. Tambe et al. [15] underlined that many different coatings exhibited poor corrosion resistance in environments with higher concentrations of anaerobic bacteria, and they tested the antibacterial properties of epoxy-based anticorrosive coatings. Hodul et al. [16] also emphasized that antibacterial coatings are often used in households where molds and bacteria are common on the walls of various buildings. Moreover, antibacterial coatings are very useful for mold prevention after flooding. Chen et al. [18] investigated the mechanical properties of antibacterial epoxy resin adhesive wood biocomposites against a skin disease, demonstrating a negative effect of mold on wooden elements in many applications and thus stressing the need for the use of antibacterial epoxy resin/wood biocomposites. Barttolozzi et al. [9] tested the mechanical and thermal properties of multifunctional copper–montmorillonite/epoxy resin nanocomposites with antibacterial activities.

The objective of this study is to demonstrate that the tested epoxy compounds can be used as adhesives for joining structural elements or as protective coatings with antiseptic properties. The addition of boric acid may contribute to increasing the safety of bonded structures containing adhesive joints or epoxy coatings in situations where the use of antiseptic materials is required (including in hospitals, schools, and commercial buildings). This literature review has also shown that boric acid can be used as a flame retardant, which further enhances the properties of such epoxy materials. This work focuses on the mechanical properties of modified epoxy compounds, an aspect which may be important when designing adhesive joints [1,6]. The use of various types of curing agents makes it possible to select epoxy materials depending on the specific requirements related to, among others, the resistance of an adhesive joint to the impact of environmental factors (e.g., temperature) or the stiffness of a produced epoxy compound.

## 2. Materials and Methods

### 2.1. Description of Analyzed Epoxy Compounds

Twelve epoxy compounds were prepared by mixing an epoxy resin with a curing agent and a modifier. The compounds were made of an epoxy resin based on bisphenol A (BPA). It is a basic epoxy resin (unmodified) with an epoxide number of 0.48–0.51 mol/100 g and an epoxy equivalent weight of 196–208.

In this study, low-temperature curing agents were used to cure the epoxy resin, which allowed for us to conduct the curing process at room temperature. The advantages of the curing process conducted at room temperature have been presented in studies by Ellis [6], Ignateko et al. [29], Hong and Tsai [54], Qaderi et al. [55], and Grillet et al. [27]. One of the advantages is that the epoxy resins cured in this way are suitable for many applications [6].

The epoxy resin was cured using one polyamide and two amine curing agents (produced by Sarzyna Resins, Nowa Sarzyna, Poland):

- a polyamide curing agent with an amine value of 290–360 mg KOH/g and a viscosity at 25 °C of 10,000–25,000 mPas (trade name: PAC, produced by Sarzyna Resins, Nowa Sarzyna, Poland)—polyaminoamide;
- an amine curing agent with an amine value of 700–900 mg KOH/g and a viscosity at 25 °C of 200–300 mPas (trade name: ET, produced by Sarzyna Resins, Nowa Sarzyna, Poland)—an adduct of aliphatic amine (triethylenetetramine) and aromatic glycidyl ether;
- an amine curing agent with an amine value of 200–350 mg KOH/g and a viscosity at 25 °C of 150–300 mPas (trade name: IDA, produced by Sarzyna Resins, Nowa Sarzyna, Poland)—an adduct of cycloaliphatic amine.

More details about the characteristics of the epoxy resin and the curing agents are given in references [5,56,57] and in Table 1 and Table 2.

**Figure 1:**
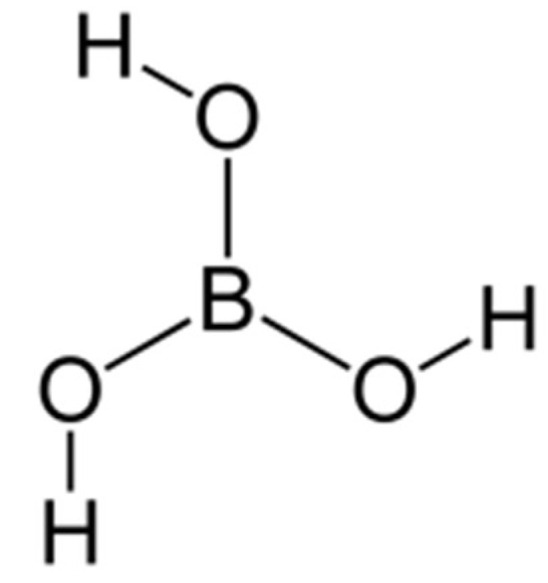
Orthoboric acid form https://pl.wikipedia.org/wiki/Kwas_borowy (accessed on 28 February 2023).

The applied resin/curing agent ratios resulted from the stoichiometric ratios of individual resins and curing agent types, as specified in Table 1. The epoxy compounds were modified by the addition of an antiseptic in the form of boric acid, H_3_BO_3_ (Chempur Company, Piekary Śląskie, Poland) in three amounts: 0.5 g; 1.0 g, and 1.5 g. Boric acid is an inorganic chemical compound with a weak acid reaction that comes in white powder form. It has a molar mass of 61.83 g/mol and a solubility in water of 4.7% (20 °C). The orthoboric acid form was used in this study (Figure 1).

The tested filler contents were selected based on the data reported in the literature [56,58] on the modification of epoxy compounds with various fillers. Studies [50,51] were conducted with the use of various amounts of fillers, including flame retardants, depending on the resin type. Also, patent descriptions (PL 230,340 B1, PL 214,525 B1) provide information about the addition of antipyrine to epoxy resin in amounts ranging from 1 to 2%. In the author’s previous studies [13,14,56], other powder fillers were added in amounts ranging from 1 to 3 (5)%. He at al. [58] showed that the functional properties of polymeric materials could be increased by adding small amounts of filler at <5 wt%. In this study, boric acid was added in three different amounts: 0.5, 1.0, and 1.5 g per 100 g of epoxy resin.

Reference samples were fabricated without the addition of boric acid. The chemical composition of the analyzed epoxy compounds and their denotations are given in Table 1. The denotations (Table 3) take into account the stoichiometric ratios of epoxy resin to curing agent in the epoxy compounds. Given the stoichiometric ratios, it was decided that the denotations indicating the contents of individual components (resin/curing agent/antipyrine) would be given in grams (Table 3).

### 2.2. Preparation of Cured Epoxy Compound Samples

**Figure 2:**
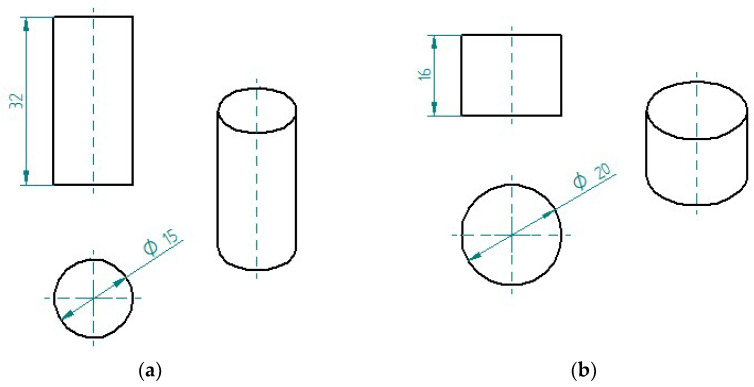
Epoxy compound samples for: (**a**) strength tests; (**b**) microscopic examination (dimensions in mm).

The samples of epoxy compounds (in the cured state) used for strength tests and microscopic examination are shown in Figure 2. For comparative purposes, the samples had the same dimensions and were produced under the same conditions, and their dimensions were selected in compliance with the specifications laid down in the standards ASTM D695-15 [59] and PN-EN ISO 604 [60]. The samples were fabricated using cylindrical polymeric molds. A total of 72 samples were fabricated for strength tests (6 samples per each of the 12 epoxy compounds—Figure 2a), and 12 samples were prepared for microscopic examination (Figure 2b).

Strength tests were conducted using a Zwick/Roell Z150 testing machine (Zwick/Roell, Ulm, Germany) according to the PN-EN ISO 604 [60]. The tests were performed with an initial force of 200 N and a test speed of 10 mm/min. The cured samples of the epoxy compounds were first mounted in a specially designed chuck and then put inside the testing machine and subjected to compression. The following strength parameters were measured: compression modulus, compressive strength, and nominal compressive strain.

Microscopic examination was performed using a digital microscope, DiGi Microscope ViTiny UM06 (ViTiny, Kaohsiung, Taiwan).

The first stage of fabricating the samples of cured epoxy compounds involved the preparation of cylindrical molds in which the samples were poured in a liquid state. The inside of the molds was sprayed with an anti-adhesion agent for 10 s from a distance of approx. 150 mm in order to facilitate the removal of the cured epoxy compounds. The anti-adhesion agent was silicon-based Soudal (manufactured by Soudal, Pionki, Poland). After that, the epoxy compounds were prepared. The ingredients of the epoxy compounds were weighed on an OX-8100 balance (manufactured by FAWAG S.A, Lublin, Poland) with a measuring accuracy of 0.1 g.

The epoxy resin was placed in a disposable container first, and then boric acid was added. Both components, i.e., the epoxy resin and boric acid, were mixed mechanically for 2 min at 730 rpm using a bench drill, and then a curing agent was added. All the ingredients were mixed mechanically in the polymer container for 2 min at a shear rate of 128 m/min until a homogeneous mass was obtained. They were mixed using a special paddle mixer. As a result, it was possible to ensure a more uniform distribution of boric acid in the prepared epoxy mass. Following the mixing process, the epoxy compounds were deaerated for 2 min with a vacuum pump.

The liquid epoxy compounds were then poured into the cylindrical molds. The modified and unmodified compounds were cured for 7 days. During curing, the air temperature ranged from 20 to 22 °C and the air humidity was 22 ± 2%. It should be remembered that once the curing agent is added to the resin, the curing process begins and there is some time left to use the mixture before it cures (open time). This time depends on several factors, such as the type of epoxy resin and curing agent, temperature, and mixture ratio, and it varies for individual conditions. For the Epidian 5 epoxy resin with the polyamide curing agent (PAC), the gelling time is 180 min at room temperature. After this time, initial curing takes place for another 6–8 h, and after 72 h, the degree of curing is approximately 80–90%. A complete curing process takes 7–14 days. The gelling time for Epidian 5 with an adduct of cycloaliphatic amine curing agent (IDA) is 40 min, while for Epidian 5 with the amine curing agent (ET), it is 30 min at room temperature. The curing time (7 days for all analyzed epoxy compounds) was selected based on the guidelines set out in the literature on epoxy resins and curing agents and the recommendations provided by the manufacturer of the tested epoxy resins and curing agents (Ciech Sarzyna, Nowa Sarzyna, Poland). The specifications regarding the mechanical properties of the cured epoxy materials given in the manufacturer’s catalogues also relate to the 7-day curing time.

The samples were removed from the molds after 7 days. After that, they were machined by milling to ensure uniform dimensions and remove surface irregularities.

### 2.3. Statistical Analysis

Obtained compressive strength results of the epoxy compounds with varying contents of boric acid used as a modifying agent were statistically analyzed using the Statistica software package (Version 13.3) by means of correlation (to determine the relationship between variables) and regression (to determine the form of the relationship). Pearson’s linear correlation coefficient r (X,Y) was adopted as a measure of the correlation between one variable and other variables. If the r value was close to 1, the examined variables were linearly related. Relevant assumptions were made for the statistical analysis, and the appropriate statistical test methods were used [61].

## 3. Results

### 3.1. Strength Test Results

Compressive Strength

**Figure 3:**
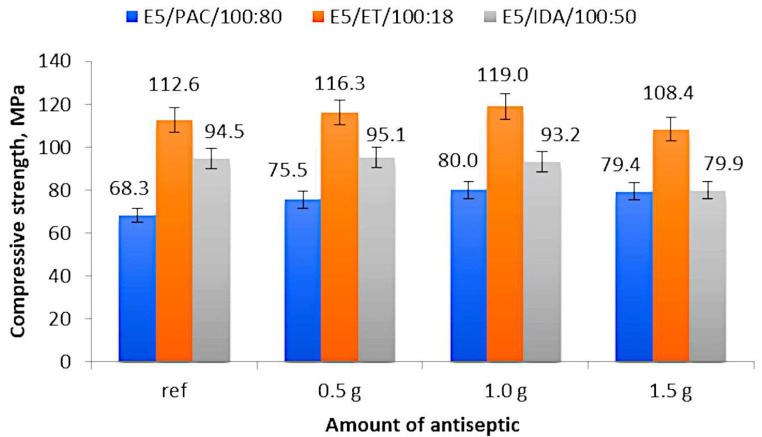
Compressive strength of modified and unmodified (reference) epoxy compound samples.

Figure 3 shows the compressive strength (average values) of 12 analyzed epoxy compounds depending on the modifier content. The figures show the average values obtained from six measurements; however, for some cases, the results were obtained from four or five measurements, which took place when the results were extremely different or when the obtained value was a gross error.

An analysis of the results for the epoxy compounds with the polyamide curing agent (Figure 3) reveals that the lowest compressive strength of 68.3 MPa was obtained for the reference samples of E5/PAC/100:80, while the highest strength of 80.0 MPa was obtained for the epoxy compound samples containing 1.0 g of boric acid (0.55% mass fraction of boric acid in the epoxy compound). A comparable strength value (79.4 MPa) was also obtained for the epoxy compound samples containing 1.5 g of boric acid (0.83% mass fraction of boric acid in the epoxy compound). The percentage difference between the highest and the lowest compressive strength amounted to 17.1%. As for the modified epoxy compounds, the lowest compressive strength (75.5 MPa) was obtained for the samples containing 0.5 g of modifier (E5/PAC/H_3_BO_3_/100:80:0.5 with 0.28% mass fraction of boric acid in the epoxy compound). This value was 10.5% higher than that obtained for the reference samples and, at the same time, it was 5% lower than the highest obtained compressive strength.

An analysis of the strength results of the epoxy compounds with the amine curing agent (ET) presented in Figure 3 demonstrates that the highest compressive strength was obtained for the epoxy compound samples E5/ET/H_3_BO_3_/100:18:1.0, containing 1.0 g of boric acid (0.84% mass fraction of boric acid in the epoxy compound). This value amounts to 119.0 MPa and was 5.7% higher than that obtained for the reference sample (112.6 MPa). The lowest compressive strength of 101.7 MPa was obtained for the samples of E5/ET/H_3_BO_3_/100:18:1.5, containing 1.5 g of boric acid (1.26% mass fraction of boric acid in the epoxy compound), and the difference between this value and that obtained for the reference samples amounted to 10.7%.

The compressive strengths of the reference sample (E5/IDA/100:50) and the sample containing 0.5 g of boric acid (0.33% mass fraction of boric acid in the epoxy compound) (E5/IDA/H_3_BO_3_/100:50:0.5) were similar and equal to 94.5 MPa and 95.1 MPa, respectively (Figure 3). The lowest compressive strength of 79.9 MPa was obtained for the epoxy compound E5/IDA/H_3_BO_3_/100:50:1.5 containing 1.5 g of antiseptic (0.99% mass fraction of boric acid in the epoxy compound), which was 19% lower than the highest obtained value. The results of the modified epoxy compounds reveal a trend that the higher the antiseptic content added, the lower the compressive strength of these compounds becomes.

**Figure 4:**
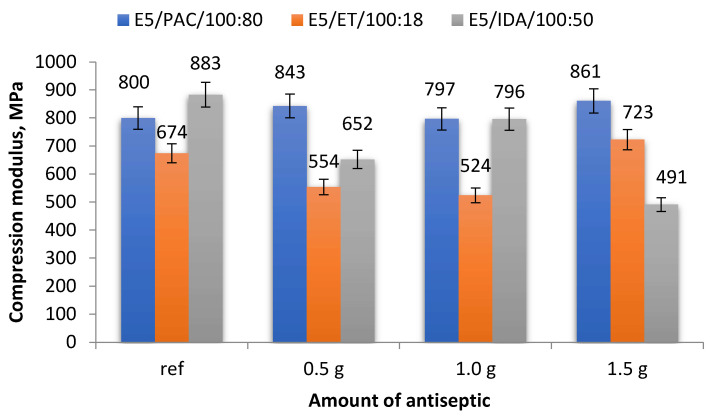
Compression modulus of modified and unmodified (reference) epoxy compounds.

A comparison of the compression moduli (average values) obtained for the analyzed epoxy compound samples, both reference and antiseptic-modified, is given in Figure 4.

A comparison of the data in Figure 4 reveals that the compression moduli of some E5/PAC/100:80 samples were similar, with their values ranging between 797 and 861 MPa. The highest compression modulus of 861 MPa was obtained for the samples containing 1.5 g of boric acid (0.83% mass fraction of boric acid in the epoxy compound), i.e., E5/PAC/H_3_BO_3_/100:80:1.5, while the lowest compression modulus (797 MPa) was obtained for the samples containing 1.0 g of modifier (0.55% mass fraction of boric acid in the epoxy compound), i.e., E5/PAC/H_3_BO_3_/100:80:1.0. The percentage difference between the highest and lowest compression moduli was 8.0%. The highest value was 7.6% higher than the compression modulus of the reference samples (800 MPa),whereas the lowest compression modulus was only 0.4% lower than that of the unmodified samples.

The strength test results presented in Figure 4 demonstrate that it is difficult to determine the effects of the modifier content on the compression modulus of the epoxy compounds cured using the amine curing agent (ET). The highest compression modulus of 723 MPa was obtained for the samples containing 1.5 g of boric acid (1.26% mass fraction of boric acid in the epoxy compound), and the difference between this value and that of the reference samples (674 MPa) amounted to 7.3%. The lowest compression modulus of 524 MPa was obtained for the samples containing 1.0 g of antiseptic (0.84% mass fraction of boric acid in the epoxy compound; E5/ET/H_3_BO_3_/100:18:1.0), and it was 28.6% lower than that obtained for the reference samples.

The highest compression modulus of 883 MPa was obtained for the unmodified epoxy compound E5/IDA/100:50 (Figure 4). The lowest compression modulus of 491 MPa was obtained for the epoxy compound E5/IDA/H_3_BO_3_/100:50:1.5, containing 1.5 g of boric acid (0.99% mass fraction of boric acid in the epoxy compound). The difference between these values was as high as 44.4%. As for the modified epoxy compounds, the highest compression modulus was exhibited by the samples of E5/IDA/H_3_BO_3_/100:50:1.0, containing 1.0 g of boric acid (0.66% mass fraction of boric acid in the epoxy compound). This value was 10.9% lower than that obtained for the reference samples.

**Figure 5:**
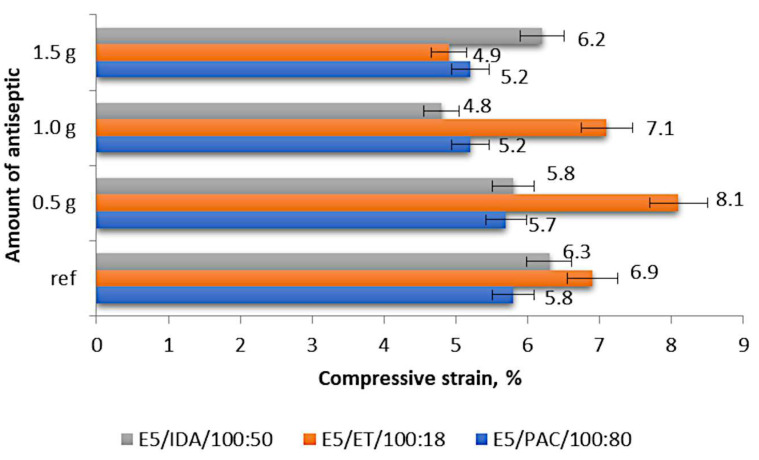
Compressive strain of the modified and unmodified (reference) epoxy compounds.

Figure 5 compares the compressive strains of the individual epoxy compounds depending on the boric acid content (in grams).

The results shown in Figure 5 demonstrate that the compressive strain of the epoxy compounds with the polyamide curing agent ranged from 5.2% to 5.8%, with the lowest values obtained for the samples containing 1.0 g of boric acid (0.55% mass fraction of boric acid in the epoxy compound; E5/PAC/H_3_BO_3_/100:80:1.0) and 1.5 g of boric acid (0.83% mass fraction of boric acid in the epoxy compound; E5/PAC/H_3_BO_3_/100:80:1.5), the latter being the highest tested modifier content. The highest compressive strain was obtained for the reference samples (unmodified epoxy compound), and the percentage difference between these values was 11.5%. Regarding the modified epoxy compounds, the compound samples containing 0.5 g of boric acid (0.28% mass fraction of boric acid in the epoxy compound; E5/PAC/H_3_BO_3_/100:80:0.5) exhibited the highest compressive strain of 5.7%. This value was 1.7% lower than that obtained for the reference samples.

The highest compressive strain of 8.1% was obtained for the epoxy compound with the amine curing agent (ET) modified with 0.5 g of boric acid (0.42% mass fraction of boric acid in the epoxy compound; E5/ET/H_3_BO_3_/100:18:0.5), as shown in Figure 5. The lowest compressive strain of 4.9% was exhibited by the epoxy compound samples containing 1.5 g of boric acid (1.26% mass fraction of boric acid in the epoxy compound; i.e., E5/ET/H_3_BO_3_/100:18:1.5). The percentage difference between these values was 39.5%. A comparison of these values with the compressive strain of the reference samples (6.9%) revealed that the highest compressive strain of the modified samples was 14% higher than that of the reference samples, while the lowest compressive strain of the modified samples was 40.8% lower than that obtained for the reference samples.

The highest compressive strain of 6.3% was obtained for the reference samples made of E5/IDA/100:50, while the lowest compressive strain amounting to 4.8% was exhibited by the samples containing 1.0 g of boric acid (0.66% mass fraction of boric acid in the epoxy compound; E5/IDA/H_3_BO_3_/100:50:1.0), as shown in Figure 5. The percentage difference between these values was 31.2%, and the difference between the compressive strain of the reference samples and the highest compressive strain of the modified samples, which was 6.2% for the epoxy compound containing 1.5 g of boric acid (0.99% mass fraction of boric acid in the epoxy compound; E5/IDA/H_3_BO_3_/100:50:1.5), was as low as 1.6%.

### 3.2. Failure Modes of Epoxy Compounds

**Figure 6:**
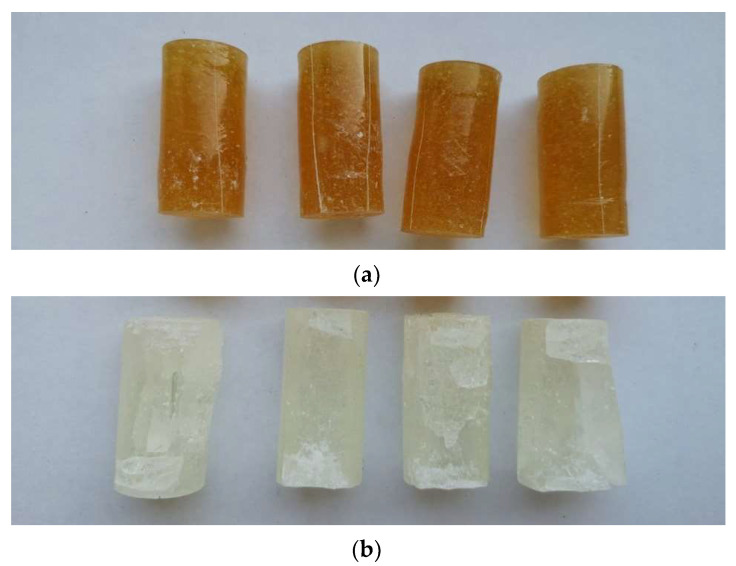
(**a**) Examples of failure modes obtained for the compressed samples of epoxy compound treated with polyamide curing agent (E5/PAC/H_3_BO_3_/100:80:0.5). (**b**) Examples of failure modes obtained for the compressed samples of epoxy compound treated with amine curing agent (E5/ET/H_3_BO_3_/100:18:0.5).

Following the strength tests, the samples of both modified and unmodified (reference) epoxy compounds were examined visually. Examples of the samples of the epoxy compounds treated with the polyamide curing agent (PAC) after failure are shown in Figure 6a, whereas Figure 6b shows the failure of the epoxy compound samples containing the amine curing agent, with the amine values ranging from 700 to 900 mg KOH/g (PF).

The samples of the epoxy compound with the polyamide curing agent shown in Figure 6 have small scratches on their circumference, but no clearly visible defects can be observed for these cylindrical samples. There are only slight shape defects that resemble buckling. More serious defects can, however, be observed on the samples of the epoxy compound containing the amine curing agent, with the amine value ranging from 700 to 900 mg KOH/g. The defects were expressed the form of mass loss, with its value being higher than that observed for the unmodified epoxy compound samples. No buckling can be observed; one can rather notice brittle fractures in some regions of these samples. There were also numerous visible cracks on the cured epoxy compound samples. In no cases, however, were the modified epoxy compound samples completely damaged. Moreover, the addition of boric acid led to a change in the color of the cured epoxy compound.

### 3.3. Optical Microscopy Results

**Figure 7:**
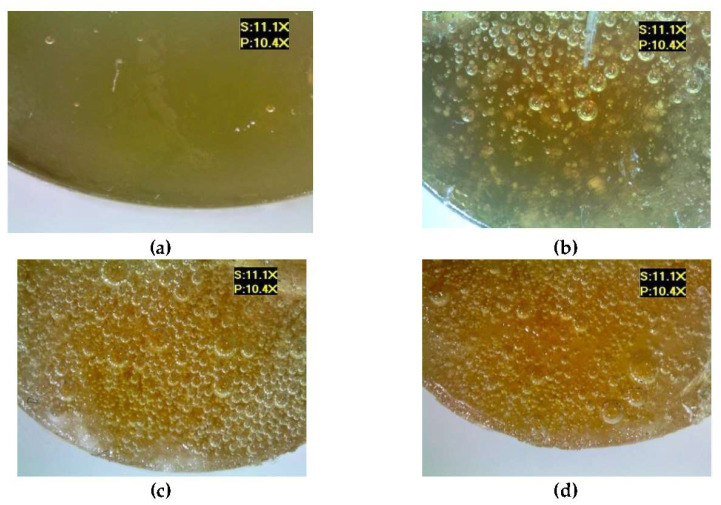
Microscopic examination results of E5/PAC/100:80: (**a**) reference, (**b**) with 0.5 g of boric acid (0.28% mass fraction of boric acid in the epoxy compound), (**c**) with 1.0 g of boric acid (0.55% mass fraction of boric acid in the epoxy compound), (**d**) with 1.5 g of boric acid (0.83% mass fraction of boric acid in the epoxy compound).

Figure 7 shows the microscopic examination results of the epoxy compounds containing the polyamide curing agent (E5/PAC/100:80), both for the reference samples and for the samples modified by the addition of different boric acid contents.

An analysis of these results demonstrates that the addition of the modifier resulted in the formation of considerably more air bubbles in the samples of the analyzed epoxy compounds. It can also be observed that the boric acid addition caused a change in the color of the compound—the sample became darker. Tambe et al. [15] also showed that the addition of modifying agents to the epoxy coating in the form of biocides caused a change in the color of these coatings, and that exposure to the SRM medium also led to changes in the microstructure and biofilm formation on the surface of the studied epoxy coatings. In this study, the modified samples also showed the presence of undissolved particles of boric acid, which indicates both the difficulty in mixing the modifier with the epoxy resin and curing agent, as well as the need to develop a correct mixing technique for the epoxy compound components. Mori et al. [21] also studied epoxy compounds containing Bisphenol A-based epoxy resins, various curing agents, and antibacterial additives (antibacterial polyelectrolyte/silver nanoparticle—Ag NP). Two types of epoxy resin were investigated to consider the effects of the resin’s chemical structure on the antibacterial activity of the filler. They also noticed that some samples had a tendency for filler particle aggregation.

**Figure 8:**
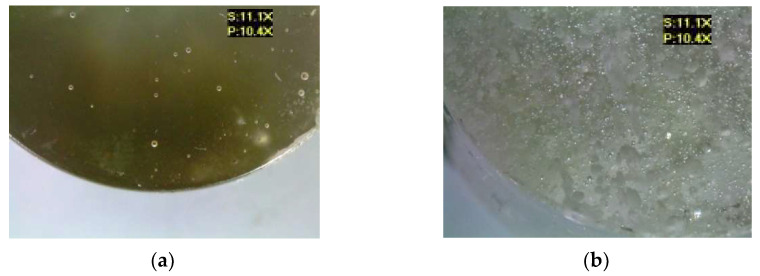

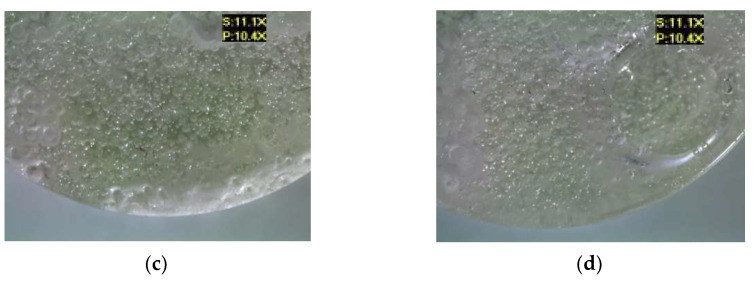
Microscopic examination results of E5/ET/100:18: (**a**) reference, (**b**) with 0.5 g of boric acid (0.42% mass fraction of boric acid in the epoxy compound), (**c**) with 1.0 g of boric acid (0.84% mass fraction of boric acid in the epoxy compound), (**d**) with 1.5 g of boric acid (1.26% mass fraction of boric acid in the epoxy compound).

Figure 8 shows the microscopic images of the E5/ET/100:18 epoxy compound, both for the reference samples and those modified by the addition of different boric acid contents.

It can be observed that the addition of the modifier led to an increase in the number of air bubbles in the analyzed epoxy compounds. The lowest number of air bubbles was observed in the reference sample E5/ET/100:15. Furthermore, the reference sample exhibited a lower surface roughness. One can also observe the presence of boric acid sediments on the lower surface of the modified samples, which may indicate that the modifier was not thoroughly mixed with the epoxy resin and curing agent, and that the modifier was prone to sedimentation.

**Figure 9:**
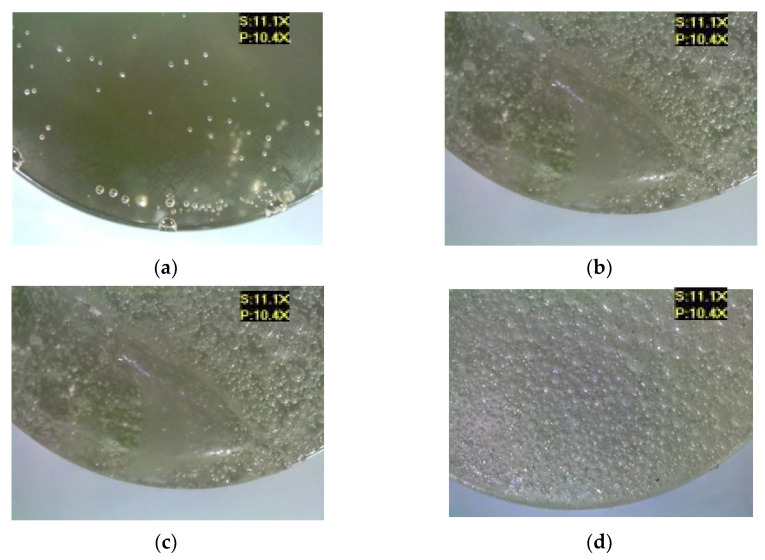
Microscopic examination results of E5/IDA/100:40: (**a**) reference, (**b**) with 0.5 g of boric acid (0.33% mass fraction of boric acid in the epoxy compound), (**c**) with 1.0 g of boric acid (0.66% mass fraction of boric acid in the epoxy compound), (**d**) with 1.5 g of boric acid (0.99% mass fraction of boric acid in the epoxy compound).

Figure 9 shows the microscopic images of the E5/IDA/100:40 epoxy compound, both for the reference samples and those modified by the addition of different boric acid contents.

The E5/IDA/100:40 epoxy compound samples showed visible particles of boric acid deposited on their lower surface. All the samples of the modified epoxy compounds showed the presence of numerous air bubbles of different sizes, which indicates that either the epoxy compound ingredients were not thoroughly mixed or that the modifier was prone to gas cavity formation.

## 4. Discussion

The strength test results did not clearly show that the addition of the antiseptic filler (boron acid) would lead to increased strength parameters of the modified epoxy compounds compared to that of the reference epoxy compounds (unmodified epoxy compounds).

The results demonstrated that the effect of the antiseptic content on the strength properties of the modified epoxy compounds depended on the curing agent type, i.e., the type of epoxy compound. It was found that the curing agent type had an impact on the mechanical properties of the modified and unmodified epoxy compounds alike (Figure 3, Figure 4 and Figure 5).

To establish a relationship between the boric acid content and the compressive strength of the epoxy compounds containing epoxy resin based on bisphenol A, a method of the linear correlation r(X,Y) between the two variables was employed. The obtained results are given in Table 4.

An analysis of the results obtained for the E5/PAC/100:80 epoxy compounds regarding the correlation between two variables, the antiseptic content (variable X) and the compressive strength of the epoxy compound (variable Y), demonstrates that the correlation coefficient r is equal to 0.906, which proves a strong linear relationship between the compressive strength of this epoxy compound type and the content of the modifying agent in the form of boric acid that was added as an antiseptic. This means that as the antiseptic content in these epoxy compounds is increased, their compressive strength increases. The coefficient of determination (r^2^) is 0.822, which means that almost 82% of the compressive strength variation can be explained by the change in the antiseptic content in the epoxy compound.

An analysis of the results for E5/ET/100:18 (Table 4) reveals that the correlation coefficient (r) for these samples is −0.278, which indicates a weak correlation between the compressive strength of this epoxy compound type and the content of the modifying agent (i.e., boric acid as an antiseptic).

Regarding the E5/IDA/100:50 epoxy compound, the correlation coefficient (r) is −0.816, which proves a strong linear relationship between the compressive strength of this epoxy compound type and the content of the modifying agent (i.e., boric acid as an antiseptic). This is a negative correlation, which means that as the antiseptic content in these epoxy compounds is increased, their compressive strength decreases.

The strength tests and the statistical analysis results demonstrate that:

- The epoxy compounds containing the amine curing agent with the amine number ranging 700–900 mg KOH/g are characterized by a higher compressive strength (Figure 3) than the compounds containing the amine curing agent with a much lower amine number (200–350 mg KOH/g) and those containing the polyamide curing agent with a lower amine number (290–360 mg KOH/g). The same trend can also be observed with respect to the compressive strain results (Figure 5);
- The differences between the compressive strength of the epoxy compounds containing the amine curing agent with a higher amine number (700–900 mg KOH/g) and the epoxy compounds with a lower amine number (200–350 mg KOH/g) depend on the boric acid content in the epoxy compounds and are as follows: 0.5 g H_3_BO_3_—18.23%; 1.0 g H_3_BO_3_—21.68%; 1.5 g H_3_BO_3_—26.29%;
- The differences between the highest compressive strength of the epoxy compounds containing the amine curing agent with a higher amine number (700–900 mg KOH/g) and the lowest compressive strength of the epoxy compounds containing the polyamide curing agent with a lower amine number (290–360 mg KOH/g) are as follows: 0.5 g H_3_BO_3_—35.08%; 1.0 g H_3_BO_3_—32.77%; 1.5 g H_3_BO_3_—26.75%;
- Although the epoxy compounds containing the amine curing agent with a higher amine number (700–900 mg KOH/g) had a higher compressive strength (Figure 3) than the compounds containing the amine curing agent with a much lower amine number (200–350 mg KOH/g), the statistical analysis results for this epoxy compound (E5/ET/100:18) showed a weak correlation between the compressive strength and the boric acid content in the sample;
- The epoxy compounds containing the polyamide curing agent show a strong positive correlation between the boric acid content in the epoxy compound and their strength. An increase in the boric acid content in the epoxy compound samples increases the strength of the epoxy compounds containing the basic epoxy resin based on Bisphenol A (BPA) with its epoxy value ranging from 0.48 to 0.51 mol/100 g.

The above observations were also confirmed by other studies [12,28,29,33], in which it was shown that the mechanical properties of various epoxy materials depended not only on the modifier content, but also on the curing agent type. Kocaman and Ahmetli [12] investigated the coating properties of modified epoxy resins of biological origins with various hardeners. Nine hardeners were used to harden the modified epoxy resins in order to compare their mechanical (tensile strength and hardness), thermal, and chemical properties. The study showed that the mechanical and coating properties depended on the curing agent type. It was also demonstrated that the tensile strength of the modified epoxy resin system was reduced compared to that of pure epoxy samples. It was also shown that the curing agent type affected the chemical resistance of those epoxy resins. Jeencham et al. [43] also showed that the addition of flame retardants improved the fire resistance and thermal stability of PP composites without deteriorating their mechanical properties. Sain et al. [33] used, among others, boric acid in combination with other fillers as flame retardants, demonstrating that the addition of the flame retardants caused a slight deterioration in the mechanical properties of polypropylene-based composites. Ignatenko et al. [29] showed that the use of aromatic amines (low-temperature aliphatic and high-temperature aromatic) as curing agents in DGEBA-based epoxy resin resulted in a very good combination of physical and mechanical properties of these epoxy materials. Ferdosian et al. [28] investigated the influence of amine hardeners (aromatic amine, DDM; aliphatic amine, DETA) on the hardening process of lignin-based epoxy resins, finding that the thermal stability of the cured epoxy resins depended on the types of curing agent and lignin used for the synthesis of the bio-based epoxy resins.

In light of the above, it can therefore be assumed that the effects of an antiseptic filler content on the mechanical properties of modified epoxy compounds depend on the epoxy compound type and, above all, on the type of a curing agent for the epoxy compound. However, regarding antiseptic properties, Coskun et al. [48] showed that the coating surfaces with the lowest concentration of NaB were characterized by a low degree of bacterial colonization, while the surfaces coated with a higher concentration of NaB (sodium borate) showed no presence of bacterial colonization or biofilm formation. This may suggest that NaB provides implanted surfaces with strong antibacterial properties.

This study analyzed the mechanical properties of epoxy compounds modified with boric acid in order to obtain materials with antiseptic properties. However, regarding the properties related to the use of boric acid as a flame-retardant additive in polymeric materials, a study by Murat Unlu et al. [36] showed that epoxy-based intumescent coatings modified with three different boron compounds, boric acid (BA), zinc borate (ZB), and melamine borate (MB), exhibited increased char yield and fire-protection properties with the addition of 1 wt% BA and MB. However, as the content of the boron compound was increased, the fire-retardant properties of the intumescent coating decreased. Doğan et al. [62] demonstrated that although the char yield increased with the boron compound content (four different boron-containing substances were analyzed: zinc borate, ZnB; borophosphate, BPO4; boron silicon containing preceramic oligomer, BSi; and lanthanum borate, LaB), the flame retarding effect was reduced. In turn, Visakh et al. [42] proved the effectiveness of using fine boric acid powder as a flame-reducing additive to epoxy resins. Hamciuc et al. [53] showed a significant reduction in the flammability and heat release capacity of epoxy-based composites containing two flame retardant additives, PFR and H_3_BO_3_, compared to a pure thermoset epoxy resin. Nazarenko et al. [43] showed that the incorporation of boric acid into the epoxy polymer matrix increased the thermal stability of the epoxy composites and led to a 2–2.7-fold reduction in toxic gaseous products.

Therefore, as emphasized by [36,60], further research must be conducted on modified epoxy materials due to the complexity of processes occurring during their modification, including the synergistic effects of various factors and a wide range of ways in which epoxy materials can be modified.

## 5. Conclusions

The results of this study lead to the following conclusions:

- The influence of the antiseptic content on the strength properties of the modified epoxy compounds depends on the type of a curing agent, i.e., the epoxy compound type;
- The curing agent type affects the mechanical properties of modified epoxy compounds. It is also worth paying attention to the differences between the tested amine curing agents, because the epoxy compounds containing the amine curing agent with a higher amine number had a higher compressive strength and strain than the compounds containing the amine curing agent with a much lower amine number. This is an interesting topic for future research;
- The compressive strength of the modified epoxy compounds did not decrease compared to that of the unmodified (reference) samples; what is more, for many cases, it even slightly increased;
- The addition of a modifier in the form of boric acid did not increase the compression modulus of the modified epoxy compounds, when compared to that of the unmodified epoxy compounds.

In conclusion, it can be claimed that the effects of antiseptic filler content on the mechanical properties of a cured modified epoxy compound depend on the type of the epoxy compound and, above all, on the type of curing agent added to the epoxy compound. It should, therefore, be emphasized that both the curing agent type and the modifying filler content should be considered when preparing epoxy compounds because the two factors may affect the strength parameters of modified epoxy compounds.

## Figures and Tables

**Table 1:** Physico-chemical properties of Epidian 5 epoxy resin [57].

Physico-Chemical Properties	
Epoxide number [mol/100 g]	0.49–0.52
Density at 20 °C [g/cm^3^]	1.17
Viscosity at 25 °C [mPas]	20,000–30,000
Epoxy equivalent weight [q/eq]	196–208
Full curing [days]	7

**Table 2:** Physico-chemical properties of curing agents [57].

Physico-Chemical Properties	Curing Agent		
	Polyamide: Polyaminoamide	Amine: Adduct of Aliphatic Amine (Triethylenetetramine) and Aromatic Glycidyl Ether	Amine: Adduct of Cycloaliphatic Amine
Amine number [mg KOH/g]	290–360	700–900	200–350
Density at 20 °C [g/cm^3^]	1.10–1.20	1.02–1.05	1.01–1.03
Viscosity at 25 °C [m·Pas]	10,000–25,000	200–300	150–300
Gelation time with Epidian 5 epoxy resin (100 g sample) at 20 °C [min]	180	30	40

**Table 3:** Types of bisphenol A (BPA)-based epoxy compounds containing boric acid.

No.	Epoxy Resin/Content	Curing Agent/Content	Modifier/Content		Denotation of Epoxy Compound
			g	%	
1	Epidian 5/100 g	PAC/80 g	-	-	E5/PAC/100:80
2			0.5 g	0.28	E5/PAC/H_3_BO_3_/100:80:0.5
3			1.0 g	0.55	E5/PAC/H_3_BO_3_/100:80:1.0
4			1.5 g	0.83	E5/PAC/H_3_BO_3_/100:80:1.5
5		ET/18 g	-	-	E5/ET/100:18
6			0.5 g	0.42	E5/ET/H_3_BO_3_/100:18:0.5
7			1.0 g	0.84	E5/ET/H_3_BO_3_/100:18:1.0
8			1.5 g	1.26	E5/ET/H_3_BO_3_/100:18:1.5
9		IDA/50 g	-	-	E5/IDA/100:50
10			0.5 g	0.33	E5/IDA/H_3_BO_3_/100:50:0.5
11			1.0 g	0.66	E5/IDA/H_3_BO_3_/100:50:1.0
12			1.5 g	0.99	E5/IDA/H_3_BO_3_/100:50:1.5

**Table 4:** Statistical analysis of the strength test results.

Variables X and Y *	r(X,Y)	r^2^	t	p	Regression Coefficient Y to X	Regression Coefficient X to Y
X—Content of antiseptic agent [g],Y—compressive strength of E5/PAC/100:80 epoxy compound [MPa]	0.906	0.822	3.036	0.009	7.560	0.190
X—Content of antiseptic agent [g],Y—compressive strength of E5/ET/100:18 epoxy compound [MPa]	−0.278	0.077	−0.409	0.007	−1.980	−0.039
X—Content of antiseptic agent [g],Y—compressive strength of E5/IDA/100:50 epoxy compound [MPa]	−0.816	0.666	−1.999	0.019	−9.140	−0.073

* X is the content of the antiseptic, Y is the compressive strength of the epoxy compound, r(X,Y) is Pearson’s linear correlation coefficient, r^2^ is the coefficient of determination, *t* is the value of the *t*-statistic examining the significance of the correlation coefficient, p is the calculated significance level for the *t*-test.

## Data Availability

Data are contained within the article.

## References

[1] Lee H.L., Neville H. (1988). Handbook of Epoxy Resins.

[2] Rudawska A., Pizzi A., Mittal K.Z. (2018). Epoxy Adhesives. Handbook of Adhesive Technology.

[3] Pertie E.M. (2006). Epoxy Adhesive Formulation.

[4] Yoon I.-N., Lee Y., Kang D., Min J., Won J., Kim M., Kang Y.S., Kim S.-H., Kim J.-J. (2011). Modification of hydrogenated Bisphenol A epoxy adhesives using nanomaterials. Int. J. Adhes. Adhes..

[5] Rudawska A., Worzakowska M., Bociąga E., Olewnik-Kruszkowska E. (2019). Investigation of selected properties of adhesive compositions based on epoxy resins. Int. J. Adhes. Adhes..

[6] Ellis B. (1993). Chemistry and Technology of Epoxy Resins.

[7] Sukanto H., Raharjo W.W., Ariawan D., Triyono J., Kaavesina M. (2021). Epoxy resins thermosetting for mechanical engineering. Open Eng..

[8] Jojibabu P., Zhang Y.X., Gangadhara Prusty B. (2020). A review of research advances in epoxy-based nanocomposites as adhesive materials. Int. J. Adhes. Adhes..

[9] Bartolozzi A., Bertani R., Burigo E., Fabrizi A., Panozzo F., Quaresimin M., Simionato F., Sgarbossa P., Tamburini S., Zappalorto M. (2017). Multifunctional Cu^2+^-montmorillonite/epoxy resin nanocomposites with antibacterial activity. J. Appl. Polym. Sci..

[10] Rudawska A., Frigione M. (2021). Cold-cured bisphenolic epoxy adhesive filled with low amounts of CaCO_3_: Effect of the filler on the durability to aqueous environments. Materials.

[11] Rudawska A. (2020). Experimental study of mechanical properties of epoxy compounds modified with calcium carbonate and carbon after hygrothermal exposure. Materials.

[12] Jin Z., Liu H., Wang Z., Zhang W., Chen Y., Zhao T., Meng G., Liu H., Liu H. (2022). Enhancement of anticorrosion and antibiofouling performance of self-healing epoxy coating using nano-hydrotalcite materials and bifunctional biocide sodium pyrithione. Prog. Org. Coat..

[13] Li W., Song B., Zhang S., Zhang F., Liu C., Zhang N., Yao H., Shi Y. (2020). Using 3-isocyanatopropyltrimethoxysilane to decorate graphene oxide with nano-titanium dioxide for enhancing the anti-corrosion properties of epoxy coating. Polymers.

[14] Dagdag O., Hamed O., Erramli H., El Harfi A. (2018). Anticorrosive Performance Approach Combining an Epoxy Polyaminoamide–Zinc Phosphate Coatings Applied on Sulfo-tartaric Anodized Aluminum Alloy 5086. J. Bio. Tribo. Corros..

[15] Tambe S.P., Jagtap S.D., Chaurasiya A.K., Joshi K.K. (2016). Evaluation of microbial corrosion of epoxy coating by using sulphate reducing bacteria. Prog. Org. Coat..

[16] Hodul J., Hodná J., Drochytka R. (2018). Antibacterial properties, shore hardness and chemical resistance of epoxy coatings containing finely ground secondary raw materials for hygienic plants. Appl. Mech. Mat..

[17] Ullah S., Ahmad F., Shariff A.M., Bustam M.A., Gonfa G., Gilani Q.F., Gillani F. (2017). Effects of ammonium polyphosphate and boric acid on the thermal degradation of an intumescent fire retardant coating. Prog. Org. Coat..

[18] Chen Z.-x., Zhang Z.-f., Aqma W.S. (2016). Mechanical characteristics of antibacterial epoxy resin adhesive wood biocomposites against skin disease. S J. Biolog Sci..

[19] Shuxia R., Huifang Y., Iushy T., Yanfang L. (2009). Preparation and properties of composite antibacterial agent. Adv. Mat. Res..

[20] Brezhnev A., Neelakantan P., Tanaka R., Brezhnev S., Fokas G., Matinlinna J.K. (2019). Antibacterial additives in epoxy resin-based root canal sealers: A focused review. Dent. J..

[21] Mori Y., Shirokawa M., Sasaki S. (2018). Antibacterial activity of epoxy resins mixed with polyelectrolyte/silver nanoparticle composite filler. Biocontrol Sci..

[22] Rudawska A. (2020). The influence of curing conditions on the strength of adhesive joints. J. Adhes..

[23] Esposito Corcione C., Freuli F., Frigione M. (2014). Cold-curing structural epoxy resins: Analysis of the curing reaction as a function of curing time and thickness. Materials.

[24] Prolongo S.G., del Rosario G., Urena A. (2005). Comparative study on the adhesive properties of different epoxy resins. Int. J. Adhes. Adhes..

[25] Mohan P. (2013). A critical review: The modification, properties and applications of epoxy resin. Polym.-Plast. Technol. Eng..

[26] Kocaman S., Ahmetli G. (2016). A study of coating properties of biobased modified epoxy resin with different hardeners. Prog. Org. Coat..

[27] Grillet A.C., Galy J., Gérard J.F., Pascault J.P. (1991). Mechanical and viscoelastic properties of epoxy networks cured with aromatic diamines. Polymer.

[28] Ferdosian F., Yuan Z., Anderson M., Xu C.C. (2015). Sustainable lignin-based epoxy resins cured with aromatic and aliphatic amine curing agents: Curing kinetics and thermal properties. Thermochim. Acta.

[29] Ignatenko V.Y., Ilyin S.O., Kostyuk A.V., Bondarenko G.N., Antonov S.V. (2020). Acceleration of epoxy resin curing by using a combination of aliphatic and aromatic amines. Polym. Bull..

[30] Brantseva T.V., Solodilov V.I., Antonov S.V., Gorbunova I.Y., Korohin R.A., Shapagin A.V., Smirnova N.M. (2016). Epoxy modification with poly (vinyl acetate) and poly (vinyl butyral). I. Structure, thermal, and mechanical characteristics. J. Appl. Polym. Sci..

[31] Shaw S.J., Ellis B. (1993). Additives and Modifiers for Epoxy Resins. Chemistry and Technology of Epoxy Resins.

[32] Yang X., Zhang Y., Chen Z., Yang Y., Jing H., Sun Z., Wang H. (2020). Preparation of epoxypropyl functionalized graphene oxide and its anticorrosion properties complexed with epoxy resin. Korean J. Chem. Eng..

[33] McDonnell G., Russell A.D. (1999). Antiseptics and disinfectants: Activity, action, and resistance. Clin. Microbiol. Rev..

[34] Chen B., Luo W., Lv J., Lin S., Zheng B., Zhang Z., Chen M. (2022). A universal strategy toward flame retardant epoxy resin with ultra-tough and transparent properties. Polym. Degrad. Stab..

[35] Hobbs C.E. (2019). Recent Advances in Bio-Based Flame Retardant Additives for Synthetic Polymeric Materials. Polymers.

[36] Murat Unlu S., Tayfun U., Yildirim B., Dogan M. (2017). Effect of boron compounds on fire protection properties of epoxy based intumescent coating. Fire Mater..

[37] Liu Z., Picken S.J., Besseling N.A. (2014). Polyborosiloxanes (PBSs), synthetic kinetics, and characterization. Macromolecules.

[38] Zhang D., Jiang N., Chen X., He B. (2020). Rheology of crosslinked entangled polymers: Shear stiffening in oscillatory shear. J. Appl. Polym. Sci..

[39] Wang S., Li Q., Wang S., Zhang W., Lu C., He X. (2023). A mechanically adaptive polymer based triboelectric nanogenerator for long-life self-powered wearable electronics. Eur. Polymer J..

[40] Bagci M., Imrek H. (2011). Solid particle erosion behaviour of glass fibre reinforced boric acid filled epoxy resin composites. Tribology Int..

[41] Jeencham R., Suppakarn N., Jarukumjorn K. (2014). Effect of flame retardants on flame retardant, mechanical, and thermal properties of sisal fiber/polypropylene composites. Compos. Part B Eng..

[42] Visakh P.M., Nazarenko O.B., Amelkovich Y.A., Melnikova T.V. (2015). Thermal properties of epoxy composites filled with boric acid. IOP Conf. Ser. Mater. Sci. Eng..

[43] Nazarenko O.B., Bukhareva P.B., Melnikova T.V., Visakh P.M. (2016). Effect of Boric Acid on Volatile Products of Thermooxidative Degradation of Epoxy Polymers. J. Phys. Conf. Ser..

[44] Paciorek-Sadowska J., Czupryński B., Liszkowska J. (2010). New polyol for production of rigid polyurethane-polyisocyanurate foams, Part 2: Preparation of rigid polyurethane-polyisocyanurate foams with the new polyol. J. Appl. Polym. Sci..

[45] Shen K.K., Hu Y., Wang X. (2019). Recent Advances in Boron-Based Flame Retardants. Flame Retardant Polymeric Materials.

[46] Shen J., Liang J., Lin X., Lin H., Yu J., Wang S. (2022). The Flame-Retardant Mechanisms and Preparation of Polymer Composites and Their Potential Application in Construction Engineering. Polymers.

[47] Sain M., Park S.H., Suhara F., Law S. (2004). Flame retardant and mechanical properties of natural fibre—PP composites containing magnesium hydroxide. Polym. Degrad. Stab..

[48] Coskun H.S., Kehribar L., Surucu S., Aydin M., Mahirogullari M. (2022). Antibacterial Effects of Sodium Borate and Calcium Borate Based Polymeric Coatings for Orthopedic Implants. Cureus.

[49] Sasany R., Eyüboğlu T.F., Özcan M. (2023). Long-Term Effect of Nanosized Boric Acid Powder on Optical Properties of Polymer Infiltrated Ceramic CAD-CAM Material. Coatings.

[50] Türkaslan B.E., Dalbeyler A. (2022). An Alternative Pre-Treatment Sterilization Solution Synthesis Utilizing Boric Acid Doped Graphene Oxide. Iran. J. Chem. Chem. Eng..

[51] Sun S., Yu Q., Yu B., Zhou F. (2023). New Progress in the Application of Flame-Retardant Modified Epoxy Resins and Fire-Retardant Coatings. Coatings.

[52] Nagieb Z.A., Nassar M.A., El-Meligy M.G. (2011). Effect of Addition of Boric Acid and Borax on Fire-Retardant and Mechanical Properties of Urea Formaldehyde Saw Dust Composites. Int. J. Carbohydr. Chem..

[53] Hamciuc C., Vlad-Bubulac T., Serbezeanu D., Macsim A.-M., Lisa G., Anghel I., Şofran I.-E. (2022). Effects of Phosphorus and Boron Compounds on Thermal Stability and Flame Retardancy Properties of Epoxy Composites. Polymers.

[54] Hong S.G., Tsai J.S. (2000). The efect of metal surfaces on the adsorption and degradation behavior of an epoxy/amidoamine system. Macromol. Mater. Eng..

[55] Qaderi S.B.A., Peck M.C., Bauer D.R. (1987). Characterization of solutions and aqueous dispersions of epoxy/amidoamine resins. J. Appl. Polym. Sci..

[56] Rudawska A. (2019). The impact of seasoning conditions on mechanical properties of modified and unmodified epoxy adhesive compounds. Polymers.

[57] Ciech Sarzyna Catalogue. https://sarzynachemical.pl/.

[58] He H., Li K., Wang J., Sun G., Li Y., Wang J. (2011). Study on thermal and mechanical properties of nano-calcium carbonate/epoxy composites. Mat. Des..

[59] (2015). Standard Test Method for Compressive Properties of Rigid Plastics.

[60] (2006). Plastics—Determination of Compressive Properties.

[61] Rabiej M. (2012). Statistics with the Statistica Program (Statystyka z Programem Statistica).

[62] Doğan M., Yilmaz A., Bayraml E. (2010). Synergistic effect of boron containing substances on flame retardancy and thermal stability of intumescent polypropylene composites. Polym. Degrad. Stab..

